# Millimeter Wave Vehicular Channel Emulation: A Framework for Balancing Complexity and Accuracy †

**DOI:** 10.3390/s18113997

**Published:** 2018-11-16

**Authors:** Thomas Blazek, Erich Zöchmann, Christoph F. Mecklenbräuker

**Affiliations:** 1 Institute of Telecommunications, 1040 TU Wien, 1040 Vienna, Austria; ezoechma@nt.tuwien.ac.at (E.Z.); cfm@nt.tuwien.ac.at (C.F.M.); 2 Christian Doppler Laboratory for Dependable Wireless Connectivity for the Society in Motion, 1040 Vienna, Austria; 3 Department of Radio Electronics, TU Brno 601 90, Czech Republic

**Keywords:** V2X communications, mmwave, channel emulation, Akaike information criterion, model order estimation, cluster channels

## Abstract

We propose a general framework for the specification of a sparse representation of millimeter wave vehicular propagation channels and apply this to both synthetic data and real-world observations from channel sounding experiments. The proposed framework is based on the c-LASSO (complex Least Absolute Shrinkage and Selection Operator) which minimizes the mean squared error of the sparse representation for a given number of degrees of freedom. By choosing the number of degrees of freedom, we balance the numerical complexity of the representation in the channel emulation against its accuracy in terms of the mean squared error. A key ingredient is the choice of basis of the representation and we discuss two options: the Fourier basis and its projection onto a given subband. The results indicate that the subband-projected Fourier basis is a low-complexity choice with high fidelity for representing clustered channel impulse responses. Finally, a sequential estimator is formulated which enforces a consistent temporal evolution of the geometry of the interacting objects in the propagation environment. We demonstrate the performance of our approach using both synthetic data and measured 60 GHz vehicular channel traces.

## 1. Introduction

Millimeter Wave (mmWave) communication systems are envisioned as integral part of the 5th generation (5G) of mobile telephony. The interest in mmWave transmission comes from the high bandwidth available, allowing for high-throughput links [1,2]. Due to the higher carrier frequency, mmWave is expected to provide highly directive transmissions, for example, through adaptive antenna arrays. These directive antennas act as spatial filters and allow for techniques such as beam steering [3] and Orbital Angular Momentum (OAM) multiplexing [4]. The predicted benefits have prompted interest in using the technology also in vehicular settings, where demands for communications are currently increasing, in both safety as well as infotainment applications [5,6,7]. These vehicular settings are well known for their challenging properties even in the sub-6 GHz band. Among these properties are large delay spreads, high non-stationarity and large Doppler spreads [8]. The challenge is even more pronounced for very high carrier frequencies [9], as the Doppler effect scales linearly with the carrier frequency [10]. This amplifies the influence of Root Mean Squared (RMS) Doppler spreads when omni-directional antennas are used [11,12,13].

For sub-6GHz communications, several benchmarking and evaluation mechanisms for Vehicle-to-Everything (V2X) networks have been established. Most prominently, network simulation tools are used to simulate the performance of the network layer [14,15]. These tools mainly analyze the protocol performance, and often assume very simple models of the underlying channel. Furthermore, measurement campaigns are often used to assess the performance [16]. While they are essential for analysis, these have the problem, especially in vehicular settings, that they lack reproducibility of the exact scenarios due to the highly dynamic nature of the vehicular channel, and the many unknown parameters, such as weather and scatterer position. Finally, as a compromise that allows reproducibility, while still capturing the physical properties and the use of real hardware, channel emulation has been used as tool to assess communication performance [17,18]. Channel emulation has proven a valuable tool, but it faces new design challenges for mmWave communications.

At sub-6GHz, ad-hoc nodes are equipped with omnidirectional antennas, at least in the plane of interest, i.e., the horizontal plane in vehicular scenarios. The channel to be modeled and emulated becomes largely independent of the equipped antennas. Conversely, due to the high directivity of mmWave communications, different antenna patterns are to be expected. Hence, the channel characteristics observed by the communicating nodes are strongly dependent on the used antenna configurations. For instance, the RMS Doppler spread depends strongly on whether beamforming is performed [13]. Thus, incorporating antennas in the emulation approach becomes a necessity at mmWave frequencies. This also means that the observed channel, when including antennas, will exhibit sparse properties. Given suitable antennas, the vehicular mmWave channel will be sparse, or show a sparse amount of clusters. This in turn lends itself very well to emulation. Therefore, analyzing the mmWave vehicular channel based on realistic measurement setups, as well as considerations of what to model are of utmost importance. On the other hand, hardware channel emulation poses a limit on the implementation complexity, hence a compromise between fidelity and complexity is required. In the following section, we give a short introduction on how these properties factor into the emulation.

### 1.1. Channel Emulation

**Figure 1:**
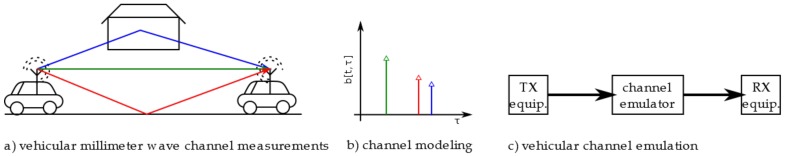
Necessary stages for channel emulation: (**a**) the encountered MPCs of a V2V transmission; (**b**) a simplified channel impulse response representation of (**a**); and (**c**) how a physical device applies the model from (**b**) to a signal going from Transmitter (Tx) to Receiver (Rx).

Channel emulation aims at following the process in Figure 1 to provide a communication channel representative of a channel vehicles encounter on the road. The propagation conditions are mapped to a signal representation, and this signal is then used in hardware to apply the modeled channel to an incoming transmission signal, and forward the resulting output to the receiver. Vehicular channel emulation can be broadly split into three categories of varying abstraction: measurement playback, geometry-based emulation and model-based emulation.

*Measurement playback* means that the hardware attempts at replaying previously recorded channel sounder data as faithfully as possible [17]. The advantage of this approach is that no statistical model assumptions have to be introduced, and uncertainty only stems from the measurements themselves, and approximations that might be necessary. The downside of the approach on the other hand is that the measured channel is not inherently representative of a larger scenario. In this case, the importance of Stage (**b**) is reduced as much as possible, ideally making it a completely transparent adaption from (**a**) to (**c**). In the case of mmWave, this is only representative for the chosen antenna setup.

*Geometry-based channel emulation* generates a vehicular channel from geometric data, employing tools such as Geometry-based Stochastic Channel Model (GSCMs) [18,19]. Alternatively, raytracing could be applied [20,21]. While these types have to make model assumptions to arrive at emulation, the benefit of this approach is that it can be executed online. As the geometry can be simulated live, an implementation that is sufficiently fast can translate the geometry to emulation at runtime, and thus provide reactive channel emulations that allow virtual drive tests. Here, Stages (**a**) and (**b**) are equally important and have to be modeled in conjunction. For mmWave, it is crucial to model the antenna position and orientation as part of Stage (**a**).

*Model based channel emulation* is based on stochastic channel models (e.g., [22]). If done correctly, such models may be representative for a whole class of scenarios, making the emulation results very interesting. Furthermore, due to the stochastic simplifications, these models tend to be of low complexity to implement in hardware. The downside of such model-based emulation is that the emulation quality is completely dependent on the model quality, and is highly challenging to adapt to more specific scenarios. For example, a typical urban channel model is usually not representative of turning a corner. Here, the emulator is completely defined at Stage (**b**), and Stage (**a**) channel measurements are only taken into account as validation and for tuning purposes, not as a model driving influence. This approach is not as strongly tied to the antenna positions as the other approaches, however very good models are needed to accurately capture the antenna effects.

In all cases, the channel modeling step differs from the an approach that is targeted towards analytical or simulation applications. Due to the necessity of hardware implementation, the model complexity, or *model order*, directly factors into the emulation step much more stringently than otherwise. Hardware channel emulators, by design, can support limited complexity, and thus every model has to be evaluated not only with respect to accuracy, but also to complexity.

### 1.2. Literature Review

Numerous measurement campaigns have studied the mmWave channel both indoors [23,24,25] and for outdoor vehicular channels [26,27,28]. These measurements frequently demonstrate a cluster structure in the impulse response. This cluster-shape was modeled by Saleh and Valenzuela [29] for wideband channels, and is frequently observed in wireless channels. Similar results are demonstrated in [30]. These results show that, while many multipaths are seen in mmWave channels, they can be clustered in a small number of scattering clusters, resulting in a cluster-sparse structure. This has been used by the authors of [31] through applying sparse methods to identify cluster positions for indoor measurements in the frequency range of 2–8 GHz. However, none of those descriptions focused on low complexity modeling aspects, or had the goal of finding representations suitable for emulation.

Channel emulation on the other hand has only been conducted on sub-6GHz channels. Results have been shown for measurement playback [17], geometric emulation [18,19], as well as model based emulation [32]. Furthermore, Field-Programmable Gate Array (FPGA) implementations are presented in [33].

Finally, very little analysis on the required model order of the mmWave channel has been conducted. The authors of [31] used sparse techniques to estimate a mmWave indoor channel, suggesting that a similar approach can work on outdoor channels. At this point, neither mmWave hardware channel emulation nor a theoretical description of the process has been published.

### 1.3. Our Contribution

In this paper, we present a framework to find a signal representation of channel sounding data that is applicable in hardware emulation setups for mmWave channels. We present a formulation based on the Complex Least Absolute Shrinkage and Selection Operator (LASSO) (c-LASSO) [34] that finds the best signal representation in the Mean Squared Error (MSE) sense under the side constraint that only *M* degrees of freedom are to be used. We present the algorithm using two basis expansions, the Fourier basis, as well as a subband-projected version of the Fourier basis. We demonstrate that the subband-projected Fourier basis is a simple, yet accurate basis to represent clustered channel impulse responses. We furthermore introduce general mechanics to implement sequential estimation which enforces geometric consistency of scattering objects [34,35]. We apply these algorithms to two sets of data. On the one hand, we use the Saleh–Valenzuela channel model [29] to demonstrate the performance of our algorithms on synthetic cluster-channel data. This analysis demonstrates that the subband-filtered Fourier basis is indeed well equipped to represent clustered channel data, especially if the multipaths are densely packed within the clusters. On the other hand, we apply the algorithms to vehicle-to-vehicle (V2V) mmWave measurements conducted in Vienna [28]. To evaluate not only the fitting performance, but also the fitting-model order trade-off, we propose to use the MSE, as well as the Akaike Information Criterion (AIC) to assess performance [36]. The AIC is an information theoretic measure that penalizes increase of the model order, to find the optimal trade-off between model order and fitting quality. Here, our results show that the clustering approach works as well as on the synthetic data, suggesting that the channel is indeed clustered. Furthermore, the AIC indicates optimal fitting trade-off at 4–6 clusters, which agrees very well with the physical interpretation of the measurement setup.

The presented framework is formulated as general as possible, and not tied to the vehicular scenario. However, our considerations and optimizations with respect to channel sparsity, clustering and scatterer mobility are targeted at the characteristics of a vehicular scenario. While this work only presents a direct approach to measurement playback, the AIC based model order evaluation provides important insights on the required complexity of stochastic models.

### 1.4. Notation

Matrices Z and vectors z are denoted by bold letters. Entries of matrices or vectors are indexed via square brackets, such as Z[0,1]. Matrix or vector size is indicated via superscripts, for example, $z^{1\times 7}$, whenever necessary. The all zeros vector (matrix) is expressed by 0, the all ones vector (matrix) is expressed by 1 and the identity matrix is expressed by I. The Euclidean norm is symbolized by ${∥\cdot ∥}_{2}$, the Manhattan norm by ${∥\cdot ∥}_{1}$, accordingly. The dagger ${\left(\cdot \right)}^{†}$ is used for pseudo inverses, ${\left(\cdot \right)}^{-1}$ is used for matrix inverses, ${\left(\cdot \right)}^{T}$ is used for transposition and ${\left(\cdot \right)}^{H}$ is used for conjugate transposition. Kronecker and Hadamard (element-wise) matrix products are denoted by ⊗ and ⊙, respectively. Diag(·) is a function that takes a vector and creates a diagonal matrix with the vector as main diagonal. Finally, peak(·,k) is a function that returns the magnitude of the *k*th largest local maximum of the first argument.

## 2. Reduced Complexity Channel Models and Their Estimation

In general, we describe a communication channel, among other descriptions, as a time-variant frequency selective transfer function h(t,f). This description is often used, since it has an immediate interpretation on how frequencies will be attenuated, and how this varies with time. In the case we consider a discrete-time system, we replace the braces by square brackets, resulting in h[t,f]. Note that, in this case, *t* and *f* are indices instead of physical quantities. They relate to the physical quantities via the sampling frequency $f_{s}$ and arbitrary offsets $f_{0}$ and $t_{0}$ as

(1) $$\begin{array}{cc} f_{\mathrm{discrete}}= & \frac{f_{\mathrm{continuous}}-f_{0}}{f_{s}}\;, \end{array}$$

(2) $$\begin{array}{cc} t_{\mathrm{discrete}}= & \left(t_{\mathrm{continuous}}-t_{0}\right)f_{s}\;. \end{array}$$

In the remainder of the paper, we only consider discrete-time representations. In this discrete-time domain, we can describe the transfer function which considers *N* frequency slots at time *t* by the vector-notation $h\left[t\right]={\left[h\left[t,1\right],h\left[t,2\right],\ldots ,h\left[t,N\right]\right]}^{T}$, with $h\left[t\right]\in C^{N\times 1}$.

The major drawback of the transfer-function description is that h[t] is usually not sparse, i.e., there are almost never any zero entries in the vector. This masks the actual required number of model parameters. For example, the studies in [37,38] demonstrate a significant improvement in channel estimation quality when using a basis that exhibits sparse properties. Hence, alternative descriptions are of interest. We introduce a general set of *N* basis vectors $a_{1},a_{2},\ldots ,a_{N}$ that we stack into a basis matrix $A\in C^{N\times N}$. Within this basis, h[t] is represented by the basis expansion b[t]

(3) h[t]=Ab[t].

Our goal is now to find a basis expansion that results in a *sparse* structure for b[t], meaning that at most *M* entries of the vector are not equal to zero. Since this is usually not possible to find exactly, we require to find a basis that minimizes a cost function e(h[t],M) given a certain sparsity order *M*. In the following two sections, we present two possible choices for such a basis.

### 2.1. Tapped Delay Line

The most standard representation besides the time-variant transfer function h[t,f] is the time-variant impulse response b[t,τ], which depends on the time and the delays. The impulse response is sparse exactly if

1. Only a small number of distinctive MPCs exist.
2. If many MPCs exist, they are grouped in a small number of *clusters*. Within these clusters, all MPCs arrive within the same sampling period, resulting in only one resolvable tap.

Then, we can describe the channel impulse response as

(4) $$b\left[t,\tau \right]=\sum_{m=1}^{M}b_{m}\left[t\right]\delta \left[\tau -\tau_{m}\left[t\right]\right]\;.$$

Here, δ[·] is the time-discrete Dirac function, and $b_{m}\left[t\right]$ and $\tau_{m}\left[t\right]$ are complex amplitude and delay of the *m*th tap, respectively. Hence, this model is called the tapped delay line model [32]. This delay line can again be written in vector notation as function of time $b\left[t\right]\in C^{N\times 1}$, and is related to h[t] via the Discrete Fourier Transform (DFT) matrix F

(5) h[t]=Ab[t]=Fb[t].

In our case, we use the unitary, centered definition of the DFT matrix with elements defined as

(6) $$A\left[n,k\right]=F\left[n,k\right]=\frac{1}{\sqrt{N}}\exp\left(-j\frac{2nk}{N}\left(n-\frac{N-1}{2}\right)\right)\;.$$

### 2.2. Clustered Delay Line

If the MPCs arrive clustered, but the sampling period is too small, the conditions in the previous sections are violated. This happens in wide-band systems, such as typical mmWave systems. Then, we see an impulse response structure such as this

(7) $$b\left[t,\tau \right]=\sum_{k=1}^{K_{0}}\sum_{l=1}^{L_{0}}b_{k,l}\left[t\right]\delta \left[\tau -\left(T_{k}+\tau_{l,k}\right)\right]\;.$$

**Figure 2:**
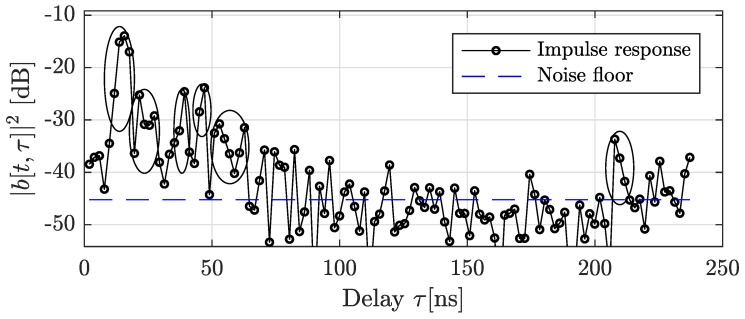
Example impulse response snapshot from measurements. The strong components clearly arrive in clusters. Potential clusters are indicated with ellipses. The noise floor is estimated via the median method.

Here, $K_{0}$ is the number of clusters, $T_{k}$ is the delay of the *k*th cluster, and $\tau_{l,k}$ is the excess delay of the *l*th tap within the *k*th cluster. The overall sparsity in this case equals $M=L_{0}K_{0}$. Of course, we can use the same basis expansion as in Section 2.1. However, we want to better exploit the cluster structure. A realization of such a cluster response seen in measurements is shown in Figure 2.

If we project the transfer function on a smaller frequency subband, we reduce the spatial resolution of our system. Thus, more MPCs will coincide on one tap, and the overall structure will be more sparse. We introduce the projection matrix $Q_{S}$, which is a diagonal matrix of the structure

(8) $$Q_{i,i}=\left\{\begin{array}{cc} 1, & i\in S\\ 0, & \mathrm{otherwise} \end{array}\right.\;,$$

with S being the block of considered subcarriers. Here, we impose the considered subcarriers to be adjacent, to achieve bandpass structure. Then, the delay line $b_{S}\left[t\right]$ which fulfills

(9) $$Q_{S}h\left[t\right]=Q_{S}Fb_{S}\left[t\right]$$

will be more sparse for smaller sets of S. We now split the full bandwidth *B* into *P* orthogonal subbands of equal size

(10) $$Q_{i,i}=\left\{\begin{array}{cc} 1 & \left(p-1\right)\frac{N}{P}<i\leq p\frac{N}{P}\\ 0 & \mathrm{otherwise}\; \end{array}\right.\;,$$

and introduce a separate basis expansion $b_{1}\left[t\right],b_{2}\left[t\right],\ldots b_{P}\left[t\right]$ for each subband $\sum_{p=1}^{P}Q_{p}h\left[t\right]=\sum_{p=1}^{P}Q_{p}Fb_{p}\left[t\right]$. Now, we introduce the concatenated vector $b_{\mathrm{subband}}$, as well as the concatenated projected DFT matrix $A_{\mathrm{subband}}$ and present a new basis expansion

(11) $$\begin{array}{cc} b_{\mathrm{subband}}= & {\left[b_{1}{\left[t\right]}^{T},b_{2}{\left[t\right]}^{T},\ldots b_{P}{\left[t\right]}^{T}\right]}^{T}\;, \end{array}$$

(12) $$\begin{array}{cc} A_{\mathrm{subband}}= & \left[Q_{1}F\;,Q_{2}F,\ldots ,Q_{P}F\right]\\ = & \left(I^{P\times P}⊗1^{(N/P)\times N}\right)⊙\left(1^{1\times P}⊗F\right)\;, \end{array}$$

(13) $$\begin{array}{cc} h[t]= & A_{\mathrm{subband}}b_{\mathrm{subband}}\left[t\right]\;. \end{array}$$

For $K_{0}$ clusters, this has a total sparseness of $M=K_{0}P$, however, as be shown below, this basis exploits the cluster structure well, and requires a smaller total model order *M* than a straightforward tapped-delay line expansion. While $K_{0}$ remains the same, we demonstrate that *P* can be chosen significantly smaller than $L_{0}$ for the same estimation quality.

The resulting estimate can be collapsed into a tapped delay line $b_{\mathrm{cluster}}$ via the Inverse DFT (IDFT)

(14) $$b_{\mathrm{cluster}}={\left(F^{N\times N}\right)}^{-1}A_{\mathrm{subband}}^{N\times NP}b_{\mathrm{subband}}\left[t\right]\;.$$

### 2.3. Sparse Channel Estimation through c-LASSO

Given a channel realization of a transfer function, caused by a sparse basis, which is corrupted by measurement noise h[t]≈Ab[t]+n[t], we estimate the sparse basis expansion $\hat{b}\left[t\right]$ using the c-LASSO [34,39], which is defined as

(15) $${\hat{b}}_{\mathrm{LASSO}}\left[t\right]=\underset{b}{arg min}\left(∥\left(h[t]-Ab\right){∥}_{2}^{2}+\mu {∥b∥}_{1}\right)\;.$$

Here, μ is a Lagrangian multiplier. The LASSO aims to minimize the MSE (via the squared 2-norm), and enforces sparse solutions through a 1-norm side-constraint.The formulation as given is a convex problem, and is thus solvable by toolboxes such as the cvx toolbox [40]. Depending on the basis A, and the magnitude of μ, a different number of nonzero parameters in different positions will be chosen for ${\hat{b}}_{\mathrm{LASSO}}\left[t\right]$. In [34], Algorithm 1 was demonstrated which finds a *M*-sparse solution given a target h, a basis A by iterating over μ until the correct sparsity order is found.

**Algorithm 1** c-LASSO algorithm.1:**procedure**c-LASSO(h,A,M)2:    w←]0,1[▹ Tradeoff between speed and convergence3:    $\epsilon \leftarrow \epsilon_{0}$▹ Threshold for detecting nonzero magnitudes.4:    $\mu \leftarrow \mu_{0}$5:    $M_{0}\leftarrow 0$6:    **while**
$M_{i}\neq M$
**do**7:        i=i+18:        $\hat{b}={arg min}_{b}\left(∥\left(h-Ab\right){∥}_{2}^{2}+\mu {∥b∥}_{1}\right)$9:        **if**
$M_{i}<M$
**then**▹ Activate additional taps10:           $u_{i}=2A^{H}\left(h-A\hat{b}\right)$11:           $U=\{m\;|\;1-\frac{u_{i}\left[m\right]}{\mu }<\epsilon \}$▹ Set of local maxima above threshold.12:           K=|U| + 113:           $\mu =\left(1-w\right)\;\mathrm{peak}\left(u_{i},K\right)+w\;\mathrm{peak}\left(u_{i},M+1\right)$14:        **else if**
$M_{i}>M$
**then**▹ Deactivate unnecessary taps15:           bisecting between μ[i−1] and μ[i−2]. ▹ For details see [34]16:        **end if**17:    **end while**18:    Refine estimate using Equation (16)19:    **return**
$\hat{b},\mu$20:
**end procedure**


When the algorithm has found a solution with the correct sparsity, the choice of $\hat{b}$ may not be optimal in the MSE sense, as the algorithm is biased by the 1-norm. To ensure that, for the resulting active set, the least-squares solution is found, the following refinement step is executed in Line 18

(16) $$\hat{b}=\left(I\left(\hat{b}\right)⊗1^{1\times M}\right){\left(A\cdot (I\left(\hat{b}\right)⊗1^{1\times M})\right)}^{†}h.$$

where I(b) is the indicator function that maps a N×1 vector to an N×1 vector with I(b)[i]=1 if b[i]=1, and I(b)[i]=0 otherwise. This computes a least squares estimate for $\hat{b}$ that only uses the *M* active taps found by the algorithm, and leaves the rest 0.

For the bisection on Line 15, Mecklenbräuker et al. [34] gave progressive approximations for efficient implementations in Equations (36)–(38). When iterating over a sequence of correlated vectors, such as time snapshots of frequency responses, the output μ should be used as initial value $\mu_{0}$ in the subsequent estimation process. In the subsequent sections, we detail several approaches at configuring the algorithm depending on the design goal.

#### 2.3.1. Configuration for Delay Line Estimation

After observing the channel response h, we now search for a *M*-sparse delay line estimate b. This is obtained directly by using F as basis A.

#### 2.3.2. Configuration for Subband Cluster Estimation

We now extend the LASSO to the subband cluster structure from Equation (13). Due to the concatenation of the *P* subband impulse responses, we now have to define in what sense we want to ensure sparseness. For this paper, we want to estimate the same level of sparseness $L_{0}$ across all subband impulse responses, resulting in a overall sparseness of $M=P\cdot L_{0}$. We achieve this by extending the Lagrangian multiplier μ to a vector μ of size P×1, and extending the LASSO as follows

(17) $$\begin{array}{cc} {\hat{b}}_{\mathrm{subband}}\left[t\right]=\underset{b_{\mathrm{subband}}}{arg min}( & ∥h\left[t\right]-\overset{A_{\mathrm{subband}}}{\overset{︷}{\left(I^{P\times P}⊗1^{(N/P)\times N}\right)⊙\left(1^{1\times P}⊗F\right)}}b_{\mathrm{subband}}{∥}_{2}^{2}\\ & +∥\left(\mathrm{Diag}\left(\mu^{P\times 1}\right)⊗I^{N\times N}\right)b_{\mathrm{subband}}{∥}_{1}). \end{array}$$

Due to the orthogonality of the projections, this formulation can be rewritten as a joint optimization of the subband impulse responses

(18) $$\begin{array}{cc} {\hat{b}}_{1}\left[t\right],{\hat{b}}_{2}\left[t\right],\ldots =\underset{b_{1},b_{2},\ldots }{arg min}\sum_{p=1}^{P} & \left(∥Q_{p}\left(h\left[t\right]-Fb_{p}\right){∥}_{2}^{2}+\mu_{p}∥b_{p}{∥}_{1}\right). \end{array}$$

In this formulation, the sum terms do not depend on each other, hence the optimization can be computed by optimizing every sum term individually. These, however, have the same structure as the c-LASSO and thus be optimized according to Algorithm 1.

#### 2.3.3. Configuration for Sequential Estimation

In the previous sections, we treat the estimation of h[t] as an optimization problem completely independent of all other times $t^{\prime }$. We refer to this as memoryless estimation. Now, we want to account for the fact that the delays in the delay lines relate to geometrical positions of scatterers. These are limited in their ability to move between two snapshots, hence the tap positions of adjacent snapshots should reflect this. However, due to noisy observations, the tap positions end up fluctuating strongly in a memoryless estimation.

Therefore, we introduce a sequential approach that tracks prior tap positions and encourages tap consistency in the estimation of the next snapshot. This is done by substituting $b=D^{-1}b^{\prime }$, and optimizing for $b^{\prime }$. As long as D is invertible, it acts as weighting matrix for the nonzero entries in b, encouraging certain taps and discouraging other [35]. This can be used to encourage consistency of tap positions, similar to [41]. The weight matrix D[0] is initialized as identity matrix as in the memoryless case. Then, iteratively, the weights are updated for new snapshots according to the following rules.

##### Propagation of Active Taps

A scatterer can physically move at most *d* delay samples between two snapshots. Thus, after calculating an estimate b[t], we adapt

(19) $$D_{j,j}\left[t+1\right]={\left(\sum_{k=j-d}^{j+d}D_{k,k}{\left[t\right]}^{-1}\right)}^{-1}$$

for all *j* that are within *d* tap of at least one nonzero entry of b[t]. This will always lower the entry in the diagonal, making it less penalized in the optimization, and thus more likely to be picked again. Here, we ensure that we do not calculate the sum across different partial impulse responses.

##### Discouraging Spawning New Taps

For all entries that were not modified in the previous step, we apply

(20) $$D_{j,j}\left[t+1\right]=D_{j,j}\left[t\right]+0.05.$$

This ensures that scarcely activated taps get progressively penalized, ensuring that random noise is less likely to activate such a tap. For the remainder of this paper, we use d=1, since we work with 2 ns delay resolution and 100μs snapshot resolution (see Section 3.2). Due to these numbers, a scatterer can physically only move one tap between snapshots.

##### Regularization

Finally, on the one hand, we want to ensure stability of the algorithm. We do this by setting the minimum value a diagonal element can take to $D_{j,j}^{\min}=0.01$. Furthermore, we set $D_{j,j}^{\max}=2$, as new taps may actually spawn. The values of these parameters can be manipulated to achieve a trade-off between algorithm convergence and geometric consistency.

## 3. Vehicular Channel Data

To demonstrate the performance of our approach, we use two sets of channel data. We first use synthetic data to test the algorithm under lab conditions. We use the Saleh–Valenzuela channel model [29] to generate synthetic clustered data that we will analyze. Furthermore, we use vehicular mmWave channel measurements to test our algorithm with actual measurements. The next two sections will give a short overview over these datasets.

### 3.1. Saleh–Valenzuela Channels

Saleh and Valenzuela published their channel model for clustered indoor channels in 1987 [29]. This model targets wideband indoor channels, and is based on the observation that the power arrived in clusters of multipaths. While the model was initially proposed for indoor settings, its focus on wide bandwidth means it has been used in mmWave channel modeling [3,42], and has also been applied to outdoor channels [43]. The channel is modeled as

(21) $$h\left[\tau \right]=\sum_{l=0}^{\infty }\sum_{k=0}^{\infty }\beta_{l,k}\exp\left(j\phi_{k,l}\right)\delta \left[\tau -T_{k}-\tau_{k,l}\right]\;.$$

Here, $T_{k}$ is the arrival time of the *k*th cluster, and $\tau_{k,l}$ is the delay of the *k*th multipath component within the *l*th cluster. The phases of all multipath components $\phi_{k,l}$ are i.i.d. uniform distributed, while all path amplitudes $\beta_{l,k}$ are Rayleigh distributed with expected power

(22) $$E\left({\left|\beta_{l,k}\right|}^{2}\right)={\left|\beta_{0,0}\right|}^{2}\exp\left(-T_{k}/\Gamma \right)\exp\left(\tau_{k,l}/\gamma \right)\;,$$

with Γ being the power decay parameters of the individual clusters, and γ the power decay parameter of the MPCs within one cluster. The delay between the (k−1)th and the *k*th cluster, as well as the delay between the (l−1)th and *l*th MPC within a cluster are exponentially distributed

(23) $$\begin{array}{cc} f(T_{k}|T_{k-1})= & \Lambda \exp\left(-\Lambda T_{k}\right) \end{array}$$

(24) $$\begin{array}{cc} f(\tau_{k,l}|\tau_{k,l-1})= & \lambda \exp\left(-\lambda \tau_{k,l}\right)\;. \end{array}$$

**Figure 3:**
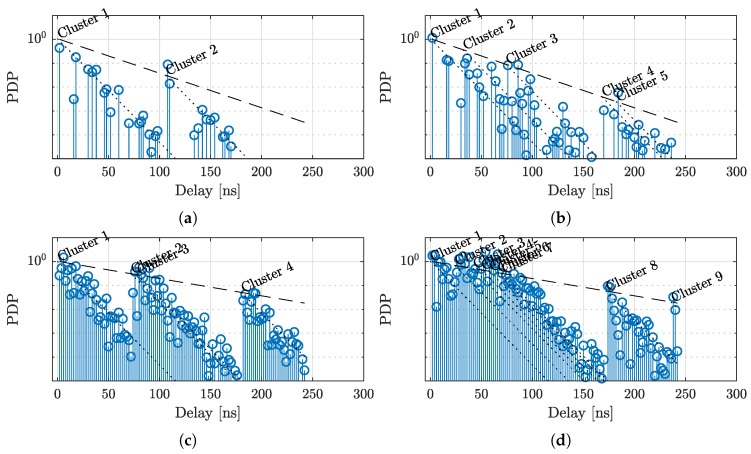
Sample Saleh–Valenzuela PDPs. The plots show a realization for Conf. (**a–d**), each produced with the same random seed. The four configurations have progressively dense intra-cluster multipaths.

We use four configurations for the channel model, that are outlined in Table 1. In Configuration (a), we directly adapt the parameters as given in [44]. Configuration (b) increases the cluster arrival rate, Configuration (c) uses the same cluster arrival rate as Configuration (b), but uses denser MPCs within the clusters, and finally Configuration (d) uses the dense clusters from Configuration (c), but increases the number of clusters even more. Sample traces for the four configurations are shown in Figure 3. The dashed line indicates the cluster power decay, while dotted lines indicate the intra-cluster power decays for the individual clusters.

### 3.2. Vehicular Channel Sounding Campaign

A detailed description of the measurement campaign and the measurement setup is found in [28]. To the benefit of the reader, we provide the key parameters in Table 2 and briefly summarize the campaign below.

**Figure 4:**
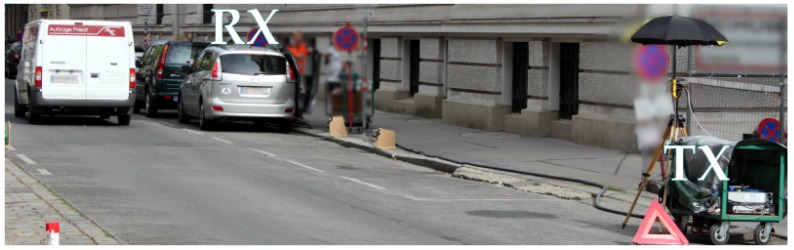
Photograph of the measurement site. The transmitter and receiver are static and urban street traffic is driving by.

To achieve very accurate frequency and time synchronization, we keep TX (a tripod) and RX (the silver Mazda) static and connected both with a 10 MHz frequency reference and a trigger cable (see Figure 4). To obtain time-variant vehicular channels, we let urban street traffic pass by. This effectively emulates a platooning scenario, where the car platoon is being overtaken. A measurement is triggered once an overtaking vehicle of the urban street traffic drives through a light barrier. The sample rate at the receiver is 600 MSamples/s. We employ a multi-tone sequence with N=121 carriers to approximately achieve a tone spacing of 5 MHz. Due to the anti-aliasing filter, we avoid the cut-off region and only transmit the sounding sequence at the $N_{s}=101$ center tones. Thereby, an effective sounding bandwidth of 510 MHz is achieved. The output of our channel sounder is the calibrated time-variant transfer function h[t,f].

**Figure 5:**
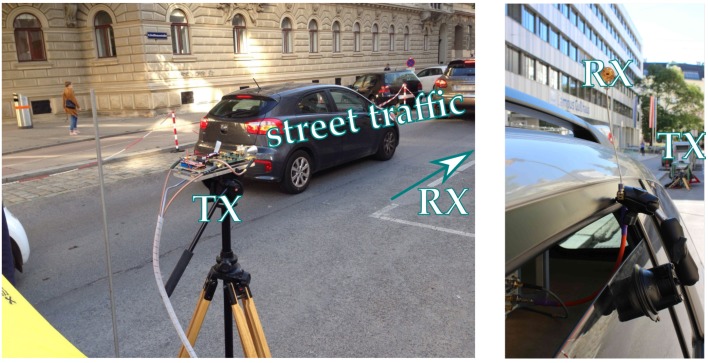
Zoomed photographs of the measurement site. On the left-hand side, the photo shows the transmit horn antenna mounted on a tripod. As multiple reflection with the transmitter car are below our receiver sensitivity, the TX car is replaced with a tripod. On the right-hand side, the photo shows the open-ended waveguide receive antenna mounted at roof height at the left, rear car side window.

We measured different overtaking vehicle types (passenger cars, Sports Utility Vehicle (SUV)s, trucks) with different transmit and receive antennas. A detailed picture of the transmitter tripod and the receive antenna mounting is shown in Figure 5.

## 4. Results

As mentioned above, in our measurements, out of N=121 total subcarriers, only the $N_{s}=101$ center subcarriers carry meaningful data. Hence, we set A=SF, with

(25) $$S=\mathrm{Diag}\left(\left[0^{1\times (N-N_{S})/2}\;1^{1\times N_{S}}\;0^{1\times (N-N_{S})/2}\right]\right).$$

This ensures that the unused subcarriers do not factor into the estimation.

### 4.1. Comparison: Peak Search

To assess our algorithm, we compare it to the performance of *peak search*. The estimate is done by calculating the dense impulse response $b=F^{-1}h$. Then, all entries except the ones with the largest *M* magnitudes are set to 0, resulting in ${\hat{b}}_{M}$. Afterwards, we refine the estimate again according to Equation (16), resulting in the *M* sparse estimate

(26) $${\hat{b}}_{\mathrm{peak}}=\left(I(\hat{b}\right)⊗1^{1\times M}){\left(F\cdot \left(I\left({\hat{b}}_{m}\right)⊗1^{1\times M}\right)\right)}^{†}h.$$

If P=1 and $D=S=I^{N\times N}$, the matrix A becomes a square orthonormal basis matrix. Under this assumption, the LASSO optimization simplifies to peak search.

### 4.2. Performance Metrics

#### 4.2.1. Absolute Estimation Quality: Mean Squared Error

We define the MSE as dependent on the bandwidth of interest, defined through S

(27) $$\mathrm{MSE}\left(h,A\hat{b}\right)=\frac{∥S\left(h-A\hat{b}\right){∥}_{2}^{2}}{N_{s}}\;.$$

Including the matrix S in the MSE is essential to fairly assess fits that are only fit to the targeted subcarriers in S.

#### 4.2.2. Balancing Model Order: Akaike Information Criterion

While the MSE is a very commonly used measure, the MSE is ill-equipped to tackle the question of over-fitting, as it does not penalize increasing the model order, and thus always favors higher model order over lower. An important measure that is able to capture the effect of over-fitting is the AIC, which is, for linear regression of Gaussian data, defined as [36]

(28) $$\mathrm{AIC}\left(h,A\hat{b}\right)=N_{s}\ln\left(\mathrm{RSS}\right)+2k\;.$$

**Figure 6:**
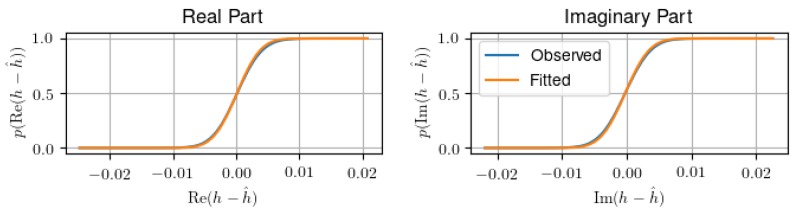
Empirical CDFs and Gaussian fits of the estimation error. The error distribution both in real and imaginary part is well approximated as Gaussian, validating the assumptions for the AIC.

RSS denotes the residual sum of squares $\mathrm{RSS}=∥S\left(h-A\hat{b}\right){∥}_{2}^{2}$, and $N_{s}$ denotes the number of estimated frequency samples. Figure 6 shows that the assumption of Gaussian data is founded, as real and imaginary part of the channel parameters are well fitted by a Gaussian distribution.

For the case where only a small number of parameters is estimated, which is usually given as $\frac{N_{s}}{k}<40$ [45], the sample-size corrected AIC is recommended

(29) $${\mathrm{AIC}}_{c}\left(h,A\hat{b}\right)=\mathrm{AIC}\left(h,A\hat{b}\right)+\frac{2k(k+1)}{N_{s}-k-1}\;.$$

In our case, every tap has two degrees of freedom (delay and complex value), hence k=2M. Thus, we use this corrected criterion as most of our analyzed configurations violate the *large parameter number* criterion.

### 4.3. Performance Analysis for Synthetic Data

Here, we analyze the performance of the c-LASSO algorithm on synthetic data given by the four configurations of the Saleh–Valenzuela model in Section 3.1. Our main focus here is how well the c-LASSO performs, depending on the *MPC density* of the clusters. Since the density of the channel is a design parameter here, the AICs optimum will be itself a function of our model parameter choices, and is of little interest. Instead, we analyze how much improvement over peak search can be achieved, depending on the chosen basis, as well as the channel model configuration. Hence, we analyze the relative change of MSE from ${\hat{b}}_{\mathrm{peak}}$ to $\hat{b}$

(30) $${\mathrm{MSE}}_{\mathrm{rel}.}\left({\hat{b}}_{\mathrm{peak}},\hat{b}\right)=\frac{|\mathrm{MSE}\left(h,A{\hat{b}}_{\mathrm{peak}}\right)-\mathrm{MSE}\left(h,A\hat{b}\right)|}{\mathrm{MSE}(h,A{\hat{b}}_{\mathrm{peak}})}\;.$$

**Figure 7:**
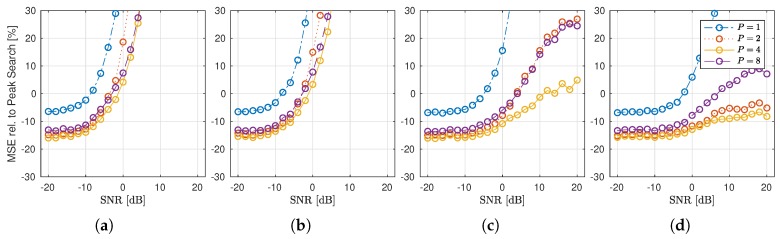
Relative change of MSE when using the c-LASSO instead of peak search. The results are shown for the four configurations (**a**–**d**) of the Saleh–Valenzuela models. The key takeaways are: (1) using P=4 shows the overall best performance across the MSE range; and (2) for very densely populated clusters, the c-LASSO noticeably outperforms peak search across a wide range of SNR (Scenarios (**c**) and especially (**d**)).

In Figure 7, this relative MSE is plotted in percent (%) from peak search to c-LASSO with P∈{1,2,4,8} for all four parameter sets over per-subcarrier SNR. All fits, both peak and c-LASSO, have been done with a total of M=16 degrees of freedom. The results show that the LASSO always outperforms peak search in the low SNR-regime, indicating that the c-LASSO works as a more robust estimator than peak search. However, of even more interest to us is, that if the channel model shows *dense* MPC clusters (Scenarios (c) and (d)), then, the performance of the c-LASSO improves in the high-SNR regime. For the subband cases of P=2,4, the algorithm uniformly outperforms the peak search by at least 10 to 15 throughout the full span. This tells us that our assumption holds, that subband-fitting is able to exploit the signal structure resulting from few MPC clusters that are densely populated to achieve better performance with equal number of degrees of freedom. Finally, the best performance is achieved at P=4, for P=8, the performance decreases again.

### 4.4. Performance Analysis for Channel Sounding Data

**Figure 8:**
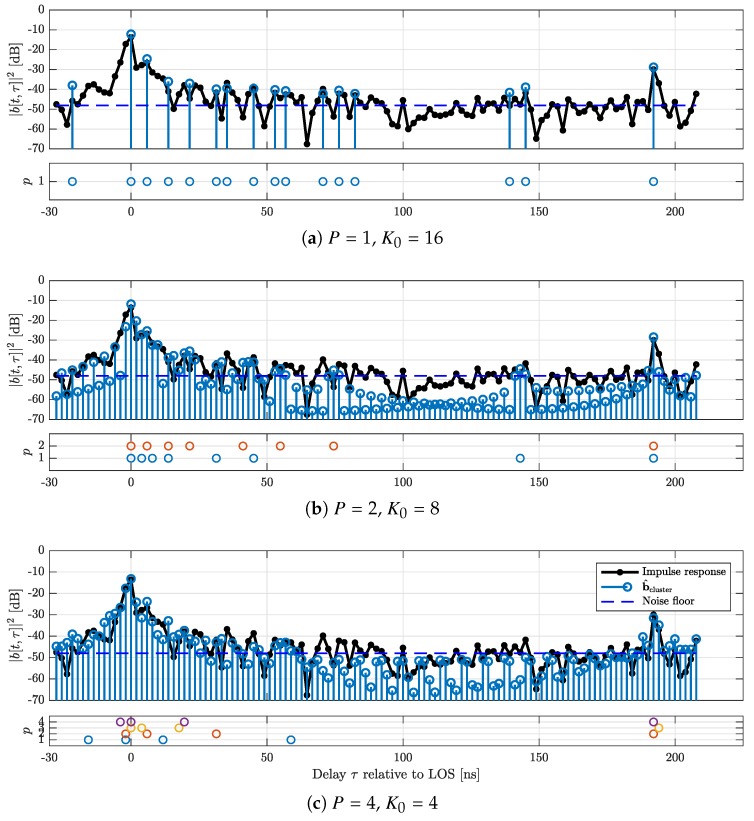
Sample impulse responses, estimated cluster impulse responses and cluster locations for M=16 and P∈{1,2,4} ((**a**); (**b**); and (**c**)).

Figure 8 shows an example result for the c-LASSO output. In black, the impulse response calculated from the original channel sounder data is shown. Furthermore, three different configurations for estimations with M=16 are displayed. For each plot, the positions of the *P* partial impulse responses are shown below the main plot. Additionally, the cluster impulse response (Equation (7)) is overlaid, as well as an estimate for the noise floor. We use the median to estimate this noise floor [46]. The plot illustrates that the impulse response shows clustered shapes, and the subband cluster approach is able to better estimate this cluster structure without expending additional degrees of freedom. The tap positions within the subband responses are highly correlated, relating to the positions of the overall clusters.

**Figure 9:**
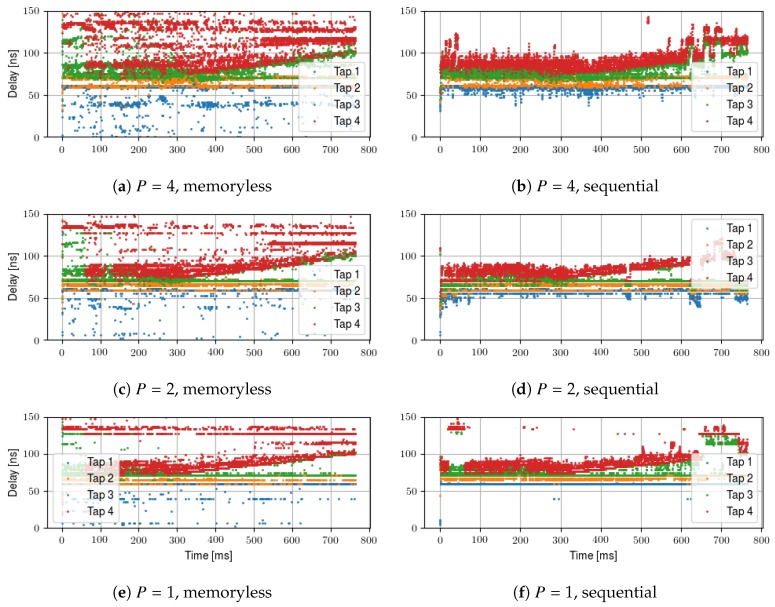
Four-tap delay evolution for an overtaking SUV: memoryless estimations (**a**,**c**,**e**); and sequential estimations (**b**,**d**,**f**). The figure shows that spatial consistency is preserved well using sequential estimation.

A time evolution of the tap positions of one measurement is shown in Figure 9. Here, the delays are color coded by arrival time, e.g., the latest estimated tap is always red. To facilitate comparison, we show one partial subband impulse response for every configuration, always with $K_{0}=4$. The results are shown both for memoryless estimation as well as sequential estimation. The results show that the memoryless estimation shows strong fluctuations of the tap positions within small time spans. Furthermore, for larger *P*, the taps tend to be spread out more. This is because of the reduced spatial resolution, which means that close taps are not distinguishable and can be represented by one tap. This allows later tap to be active more likely, as the cluster close to the LOS path collapses and does not use additional taps. In comparison, the sequential estimation effectively reduces the position fluctuations. The taps can still fluctuate in short time spans. For example, a new MPC cluster appears in Figure 9 at t≈550 ms, τ≈120 ns. At that moment, the fixed amount of taps will be redistributed, and hence the positioning appears to “jump”. However, the overall spatial consistency is drastically improved.

**Figure 10:**
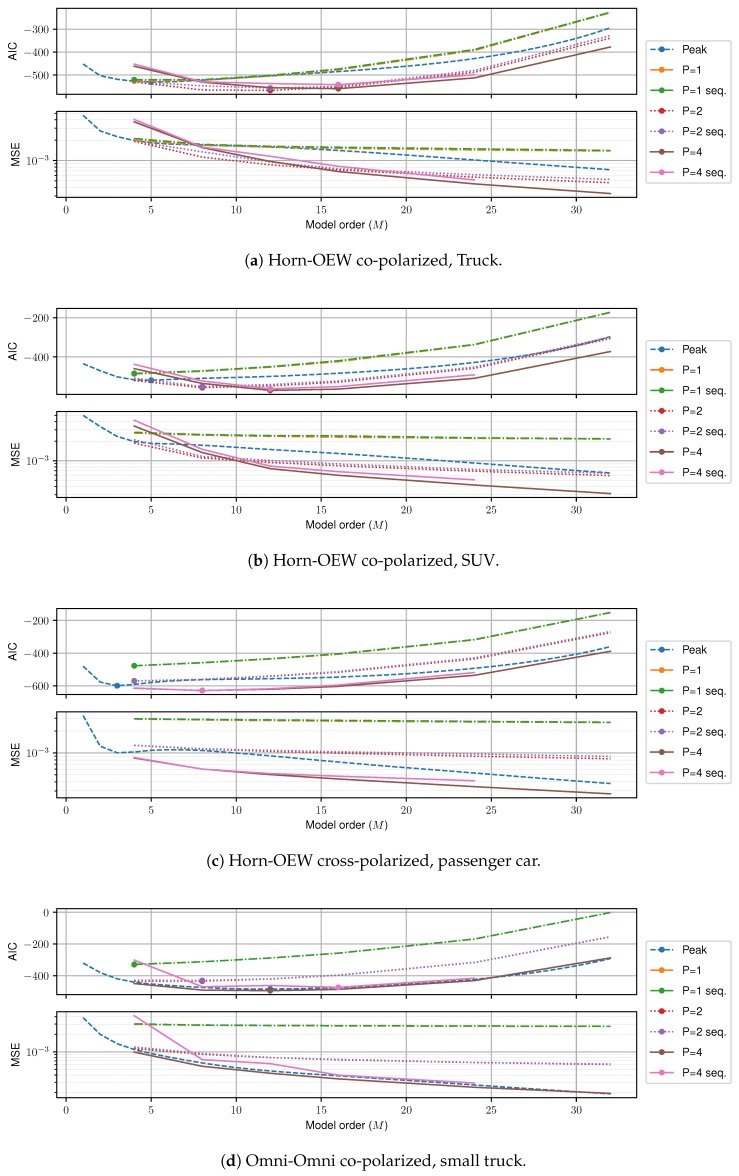
MSE and AIC of the analyzed measurements (**a**–**d**).

Finally, Figure 10 shows the MSE and AIC for four different measurement runs. We first consider the fair comparison of the memoryless case with respect to *M*. As shown in Figure 10, P=4 memoryless is uniformly the best estimator for all *M* and all measurements. In the case of the Horn-to-Open-Ended Waveguide (OEW) measurements, the gain over peak search is sizeable. Here, the spatial filtering leads to a low number of total clusters, meaning that large gains can be achieved by applying the c-LASSO. On the other hand, for the Omni-to-Omni measurements, the least amount of spatial filtering is applied, and conversely the c-LASSO estimation suffers. However, even for this worst case, the c-LASSO performs at least as well as peak search. In terms of modeling trade-off, the AIC shows a clear minimum (depicted by dots) for all models. The optimal trade-off in terms of AIC is always achieved by the P=4 memoryless c-LASSO at *M* between 8 and 16.

Sequential estimation introduces a small MSE-penalty, which is due to the additional side-constraint that we introduced. This penalty is generally very small, and the P=4 sequential c-LASSO is the second best LASSO estimator by a large margin, and only for the Omni-to-Omni setup, peak search slightly outperforms the LASSO estimator. However, this comparison is not entirely fair, as peak search never considers spatial consistency.

### 4.5. Discussion: Number of Observed Clusters during the Measurements

**Figure 11:**
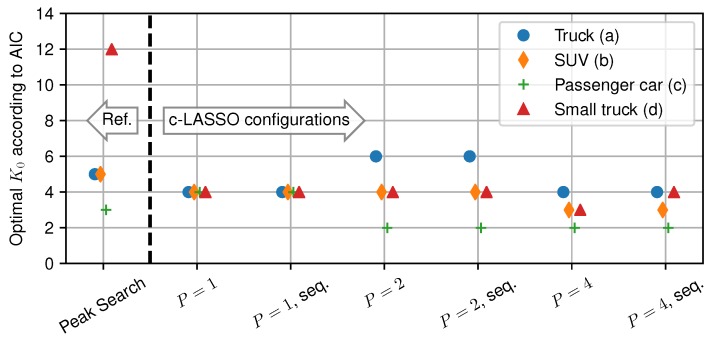
Optimal number of clusters $K_{0}$ for all presented estimation basis and measurements. To the left of the separator, peak search is given as reference. On the right, different c-LASSO configurations are shown. The numbering is consistent with Figure 10.

Now, we use the AIC to estimate the number of relevant scattering clusters. To analyze this, we take the optimal *M* from Figure 10, and calculate the number of clusters $K_{0}$ from it. The results of this for all measurements and estimation configurations are shown in Figure 11. On the left, the results for peak search are shown, while, on the right, optimal parameters for different c-LASSO settings are demonstrated. Using the normalization with respect to $K_{0}$, we clearly see that all estimation configurations have a consistently small number of relevant multipath clusters. All c-LASSO configurations have the optimal trade-off for $K_{0}=2$–6 clusters, with the large truck contributing the most clusters. Using the normalization with respect to $K_{0}$, our results show that for the given measurement setup, we never require more than at most six multipath clusters $K_{0}$.

## 5. Conclusions

When considering emulation of vehicular channels, implementation complexity is always a necessary consideration. Hence, finding a suitably sparse representation of the communication channel is mandatory for high fidelity channel emulation. Due to the large bandwidth in mmWave communications, and the size of vehicles on the road, clustered channel impulse responses are to be expected. If this cluster structure is exploited in the channel modeling approach, the resulting model is shown in this paper to exhibit a sparse structure. Thus, this paper provides a general framework of finding a sparse representation of channel data, by using the c-LASSO with a suitable basis. The optimal choice of the basis matrix A depends on the properties of the channel, as well as implementation limits of the emulator. The subband-projected Fourier basis is able to estimate both the measured channel as well as the synthetically generated channel with a high fidelity while keeping the total number of degrees of freedom low. To quantify the optimal trade-off, we apply the AIC as an effective measure for the optimal number of degrees of freedom that should be spent on a given channel. Our results show that fitting six multipath clusters is optimal in the accuracy–complexity trade-off. While this number can change depending on the scenario, it provides a good indicator of required complexity. If complexity allows it, coupling this with P=4 parallel subbands has proven an overall optimal complexity in the AIC sense. Our results are consistent with the cluster assumption, as well as the overall geometry of our measurements. Finally, the presented framework itself is agnostic to the type of used channel, and not tied to V2X scenarios.

## Figures and Tables

**Table 1:** Saleh–Valenzuela parameter combinations.

Paramter unit	Unit	Symbol	Conf. (a) [44]	Conf. (b)	Conf. (c)	Conf. (d)
Cluster energy decay	ns	Γ	60	60	120	120
Cluster arrival rate	ns ${}^{-1}$	Λ	1/300	1/90	1/90	1/30
MPC energy decay	ns	γ	20	20	20	20
MPC arrival rate	ns ${}^{-1}$	λ	1/5	1/5	5	5

**Table 2:** Channel sounding measurement parameters. For the full parameter set, refer to [28].

Parameter	Value
Center frequency	60 GHz
Subcarrier spacing	4.96 MHz
Number of subcarriers	102
Snapshot rate	129.1 μs
Delay resolution	1.96 ns
Recording time	720 ms

## Abbreviations

The following abbreviations are used in this manuscript:

**AIC** Akaike Information Criterion
**c-LASSO** Complex LASSO
**CDF** Cumulative Distribution Function
**DFT** Discrete Fourier Transform
**FPGA** Field-Programmable Gate Array
**GSCM** Geometry-based Stochastic Channel Model
**IDFT** Inverse DFT
**LASSO** Least Absolute Shrinkage and Selection Operator
**mmWave** Millimeter Wave
**MPC** Multipath Component
**MSE** Mean Squared Error
**OAM** Orbital Angular Momentum
**OEW** Open-Ended Waveguide
**PDP** Power Delay Profile
**RMS** Root Mean Squared
**SNR** Signal-to-Noise Ratio
**SUV** Sports Utility Vehicle
**V2V** Vehicle-to-Vehicle
**V2X** Vehicle-to-Everything
